# The Impact of Different Types of Hydrocarbon Disturbance on the Resiliency of Native Desert Vegetation in a War-Affected Area: A Case Study from the State of Kuwait

**DOI:** 10.3390/plants10091945

**Published:** 2021-09-18

**Authors:** Eman Kalander, Meshal M. Abdullah, Jawad Al-Bakri

**Affiliations:** 1 Department of Land, Water and Environment, School of Agriculture, The University of Jordan, Amman 19328, Jordan; emahk@hotmail.com; 2 Geography Department, College of Arts and Social Sciences, Sultan Qaboos University, Muscat P.O. Box 50, Oman; 3 Department of Ecology and Conservation Biology, Texas A&M University, College Station, TX 77843, USA; 4 Natural Environmental Systems and Technologies (NEST) Research Group, Ecolife Sciences Research and Consultation, Hawali 30010, Kuwait

**Keywords:** arid ecosystem, burning oil field, Kuwait, total petroleum hydrocarbon (TPH), soil contamination

## Abstract

This study assesses the impact of total petroleum hydrocarbon (TPH) concentration and soil parameters (heavy metals, chemical properties, and water-soluble boron) on the succession process of vegetation survival in the Al-Burgan oil field in Kuwait. A total of 145 soil samples were randomly collected from the three main types of hydrocarbon contamination, including dry oil lake (DOL), wet oil lake (WOL), and tarcrete. Sampling was also extended to noncontaminated bare soils that were considered reference sites. Remote-sensing data from Sentinel-2 were also processed to assess the level of contamination in relation to soil surface cover. The results showed that TPH concentration was significantly higher in WOL and DOL (87,961.4 and 35,740.6 mg/kg, respectively) compared with that in tarcrete (24,063.3 mg/kg), leading to a significant increase in soil minerals and heavy metals, greater than 50 mg/kg for Ba, and 10 mg/kg for V, Zn, Ni, and Cr. Such high concentrations of heavy metals massively affected the native vegetation’s resiliency at these sites (<5% vegetation cover). However, vegetation cover was significantly higher (60%) at tarcrete-contaminated sites, as TPH concentration was lower, almost similar to that in uncontaminated areas, especially at subsurface soil layers. The presence of vegetation at tarcrete locations was also associated with the lower concentration of Ba, V, Zn, Ni, and Cr. The growth of native vegetation was more likely related to the low concentration of TPH contamination at the subsurface layer of the soils in tarcrete sites, making them more suitable sites for restoration and revegetation planning. We concluded that further investigations are required to provide greater insight into the native plants’ phytoextraction potential and phytoremediation.

## 1. Introduction

Hydrocarbons and heavy metals are globally a common source of soil pollution. Although heavy metals are naturally present in the soil, geologic and anthropogenic activities increase the concentrations of these elements to amounts that are harmful to plants, animals, and human [1,2]. Increasing the soil concentration of heavy metals can negatively impact physical, chemical, and geotechnical soil properties, leading to significant changes in physiological and biochemical processes of plant growth including the major soil elements and soil grain size [3,4].

During the second Gulf War in 1990, various ecosystems in Kuwait were impacted and contaminated by hydrocarbons. Hydrocarbon contamination is unique and is one of the worst environmental disasters of recent times. Around 6–8 million barrels of crude oil were spilled into the marine and terrestrial environment, and approximately 2–3 million barrels of crude oil were burnt and released into the environment [5,6]. As a result of such hydrocarbon disturbance, three main types of hydrocarbon contamination are present in Kuwait’s desert, namely, wet oil lake (WOL)**,** dry oil lake (DOL), and tarcrete-contaminated sites. Oil lakes are accumulations of spilled crude oil from damaged well-heads and pipelines in topographically low-lying sites within the oil fields. They are differentiated into WOL and DOL [7,8]. Tarcrete-contaminated sites consist of oil mist (oil rain) and oil soot, and occur within the upper layers of the soil in the form of an unconsolidated soil layer with a thickness of 2–8 mm. Soil contaminated by tarcrete is estimated to be in the order of 6% of Kuwait’s total area [7]. 

Thirty years after the second Gulf War, these hydrocarbon contaminations are still present in Kuwait, covering large sites. The contaminated sites are currently included in the restoration and remediation program. Previous studies found that some native plants in Kuwait can survive and grow over hydrocarbon-polluted soils, including the perennial shrubs *Haloxylon salicornicum* and *Rhanterium eppaposum*, and the perennial sedge *Cyperus conglomeratus* [9]. The regrowth of native desert plants may also vary from one site to another, depending on the extent of disturbance [10,11]. Vegetation growth was slower at some oil-contaminated areas, such as Umm Gudair and the Sabah Al Ahmad Nature Reserve, in the first two years (1991–1993) due to the higher degree of contamination at these sites. 

The recovery of such native desert plant communities could be associated with the soil type, geomorphological features, and total petroleum hydrocarbon (TPH)-contamination at the sites. Vegetation regrowth was higher at Petrocalcid soils in Umm Gudair sites [12], which store high quantities of water, potentially making it available for plant uptake during dry periods [13]. Petrogypsid soils also show good potential for vegetation growth at Wadi Al Batin [12]. The regrowth of some native desert plants was observed over a layer of clean sediment covering the oil layers, as massive remobilization of sand sheets was found in the oil-affected areas (Koch and Le-Baz 1998). However, some native vegetation could recover directly over TPH-contaminated soils. 

The recovery speed of phytoremediation over TPH contaminated lands mainly depends on the type of oil contamination and the plants. Previous studies illustrated that developing a better understanding of the mechanisms underlying the accumulation of heavy metals in plants will improve phytoremediation efficiency. The main processes in phytoremediation include the mobilization of heavy metals, root uptake, xylem loading, root-to-shoot transport, and sequestration [14]. Studies on contamination by TPH examined the growth of native desert vegetation over hydrocarbon contamination without assessing the level of resilience of both soils and plants over different types of TPH contamination and heavy metals. Therefore, this study assesses the existing levels of TPH and soil chemical properties among the three different types of TPH-contaminated site, and the impact on the succession process of vegetation survival and growth in the Al-Burgan oil field in Kuwait. The study also assesses the concentration of soil parameters and levels of vegetation cover as indicators of resilience level in TPH-contaminated sites. 

## 2. Methods and Materials

### 2.1. Study Area 

**Figure 1:**
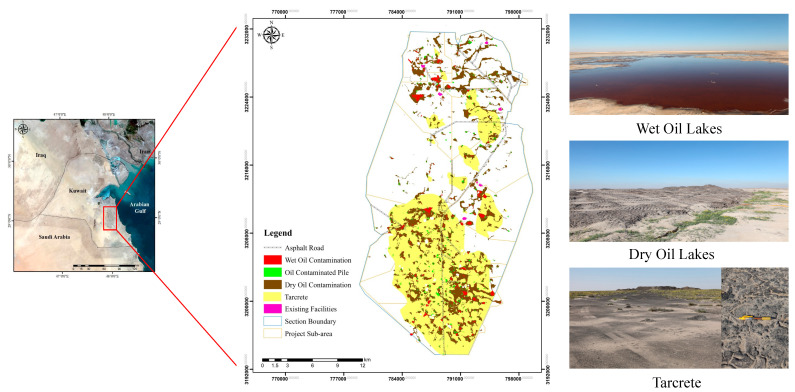
Al-Burgan oil field in southeast Kuwait, showing different types of hydrocarbon contamination: tarcrete, dry oil lakes, and wet oil lakes (KOC, 2019).

This research was conducted at the Al-Burgan oil field located in the southeast of Kuwait (Figure 1). This site is currently damaged and is subject to the restoration and revegetation program [9]. It was an oil-extraction site before it was destroyed. Al-Burgan oil field is considered to be an ideal site for this study, as it accounts for 40% of the total contaminated landscape with approximately 90% of 35.4 km^2^ oil lakes formed in this site [15]. It also covers all types of TPH contamination, including wet oil lake (WOL), dry oil lake (DOL), and tarcrete. It is the world’s largest sandstone oil field with a total surface area of about 1000 km^2^ subdivided into the Burgan (500 km^2^), Magwa (180 km^2^), and Ahmadi (140 km^2^) sectors (Kaufman et al., 2002). Figure 1 represents the land-use map of Al-Burgan oil field, showing all activities, including the boundaries of the different types of oil contamination. The map was generated by the Kuwait Oil Company (KOC) in 2019. 

The study area has a desert climate that dominates Kuwait, with dry summers and a short warm winter, and winds of dust blowing primarily during the summer months. The rainfall season usually begins in October and ends in May, with an average rainfall of 120 mm [16]. The mean air temperature is about 26.8 °C with mean maximal and minimal air temperatures of 34.1 and 19.7 °C respectively. Maximal air temperature may reach up to 50 °C in the summer, while the minimal air temperature is about 18 °C in the winter [17]. 

**Figure 2:**
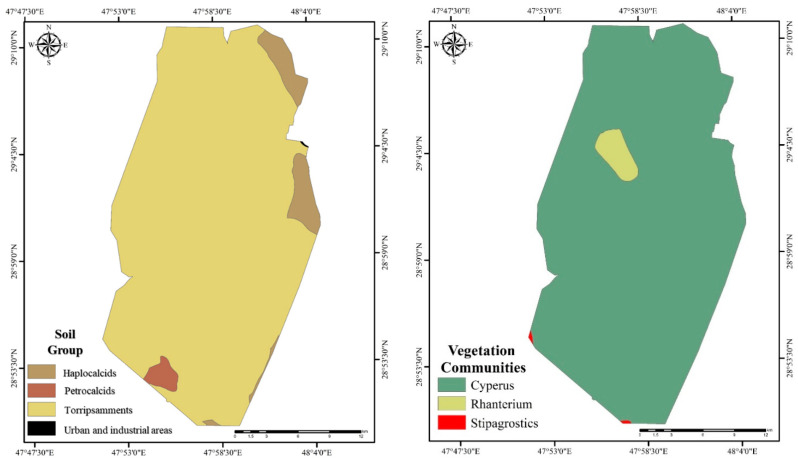
Soil group and vegetation community maps of Al-Burgan oil field [17,18].

Most of southern Kuwait is covered with sand sheets and sand dunes with isolated calcareous sandstone outcrops, and the parent material has sand- or gravel-including carbonate degrees with gypsum. According to the soil taxonomy, the study area is dominated by Torripsamment soils, followed by Haplocalcids and Petrocalcids, which cover small portions of the study site (Figure 2) [18]. The soil in the study area is characterized by extremely low permeability ((1.15–10) × 10^−6^ m/s) due to the presence of the oil deposit at the top layer (Al-Duwaisan et al., 2011). The study site is dominated by the *Cyperus* plant community, with *Rhanterium*, and *Stipagrstics*, covering small portions of the study site [17]. 

### 2.2. Data Collection 

#### 2.2.1. Soil Data Collection

A total of 145 soil samples were collected by the Kuwait Oil Company (KOC) using a random sampling scheme that covered WOL, DOL, tarcrete, and bare soils as reference sites. Samples were analyzed by Kuwait Certified Lab Company (KCL) and Kuwait International Laboratory (LABCO). The number of samples differed between the examined hydrocarbon contamination categories in the range of 25–51 samples for each category, including the surface and subsurface layers of soil. The procedure for subsurface soil sampling also differed between the three categories (Table 1). WOL and DOL samples were collected from the surface and subsurface layers down to a depth of 1.85 and 1 m, respectively. For tarcrete-contaminated sites, samples were collected from the surface and subsurface layers to a depth of 0.15 m. Standard methods of sampling and analysis were followed for all sites, as shown in Table 2. 

#### 2.2.2. Remote-Sensing Data Collection and Processing

Sentinel-2A multispectral satellite images were downloaded from the Copernicus Open Access Hub website (https://scihub.copernicus.eu/, accessed on 12 November 2020) to cover the study site. Images were acquired in February 2019 during the vegetation growing season. Multispectral data provided 12 spectral bands, 4 bands of high resolution (10 m), and 5 days of geometric revisiting [19], which are suitable for vegetation-monitoring studies [20,21]. The visible bands, including the blue (B), green (G), and red (R), were stacked together for the visual characterization of polluted sites, while the normalized difference vegetation index (NDVI) was derived from the near-infrared (NIR) and R bands at a spatial resolution of 10 m using the following equitation:

(1) $$\mathrm{NDVI}=\frac{\mathrm{NIR}\;\left(B8\right)-R\;\left(B4\right)}{\mathrm{NIR}\;\left(B8\right)+R\;\left(B4\right)}$$

where NIR is the near-infrared spectral band; R is the red spectral band; and B4–B8 are band labels. 

At this spatial scale, the NDVI can detect vegetation in arid sites well [22]. Thus, the NDVI layer was stacked with the red, blue, and green bands, and region-of-interest (ROI) polygons were collected from the vegetation and bare soils. Afterwards, ground-truth points were collected from the classes to be used in the accuracy assessment using ENVI software. Then, the support-vector-machine (SVM) classification method was implemented to determine the vegetation cover, as it had shown high accuracy in previous studies. Lastly, the vegetation coverage at each TPH contamination category was calculated on the basis of the classified layer by using the shapefile layer for the TPH contamination categories. This was performed by multiplying the counted pixels by the pixel size of the Sentinel imagery (10 × 10 m) and converting the result into a percentage (%) on the basis of the total area for each category layer using the Spatial Analyst tool in ArcMap 10.7.1 software.

#### 2.2.3. Assessing Impact of TPH and Soil Parameter Concentrations on the Resiliency of Native Plants 

To determine differences within the three types of hydrocarbon contamination, analysis of the statistical variance test within JMP™ statistical software was utilized. Then, simple linear-regression analysis tests were used to understand the relationship between the soils’ TPH and parameter concentrations. This was followed by a forward stepwise regression analysis test to determine the optimal model that combined multiple factors of soil parameters with the TPH concentration. Lastly, the vegetation-cover percentage for each TPH-contaminated category was compared with the three TPH categories and the soil parameters to determine the impact of soil heavy-metal and nutrient concentrations on plant growth. 

## 3. Results

### 3.1. Concentration of Hydrocarbon Contamination

**Figure 3:**
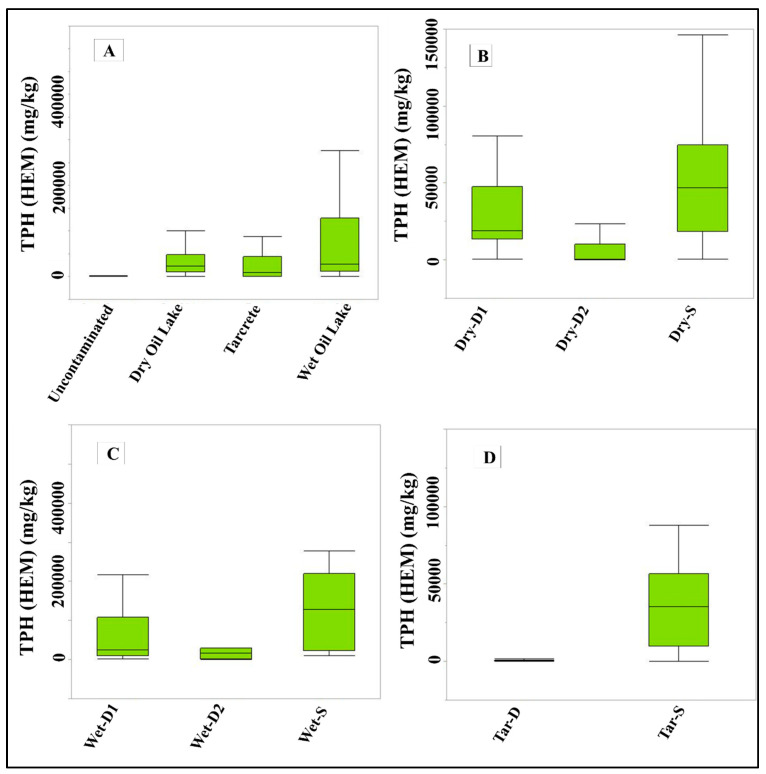
Results of TPH analysis of soil samples for (**A**) all four categories, (**B**) DOL, (**C**) WOL, and (**D**) tarcrete for different soil depths.

The results showed that hydrocarbon contamination differed between bare soils and the three oil disturbance types, with TPH concentration being significantly higher at the TPH-contaminated sites than in the bare soil (Table 3). The level of hydrocarbon disturbance also varied between the three major types of hydrocarbon disturbance (Figure 3A). The highest concentration of TPH occurred in the WOL, reaching a mean value of 87,961 mg/kg, compared with DOL, where the mean value was 35,740 mg/kg, and tarcrete, where the mean value was 24,063 mg/kg. The three sites showed variable levels of contamination when compared with the sites of bare soils, where the TPH mean value was 726 mg/kg. Some similarities in soil parameters were determined between WOL and DOL compared with the tarcrete. 

Significant differences were also observed between the surface and subsurface layers of soil for the three oil categories, as shown in Figure 3. In WOL, the concentration of TPHs differed between the surface (0.0–30 m depth) and subsurface soil layers: the concentration of TPHs at the surface soil reached 155,487 mg/km, whereas it was 69,848 mg/kg for the subsurface layer at a depth of 0.31–0.65 m. Subsurface soil layers were associated with a higher number of aliphatic aromatics C35–C90 (Figure 3). In DOL, a lower amount of TPH concentration was found in both surface and subsurface soils. TPH concentration reached a mean value of 53,803 mg/km in the surface soils and 30,695 mg/kg in the subsurface soils. Tarcrete presented the lowest TPH concentration in the surface and subsurface soils compared with WOL and DOL. TPH concentration was lower in the subsurface soils, with a mean value of 37,889 mg/km for the surface and 664 mg/kg in the subsurface soils, showing a similar TPH concentration at uncontaminated sites in subsurface soils. 

### 3.2. Impact of Hydrocarbon Contamination on Soil Properties

**Figure 4:**
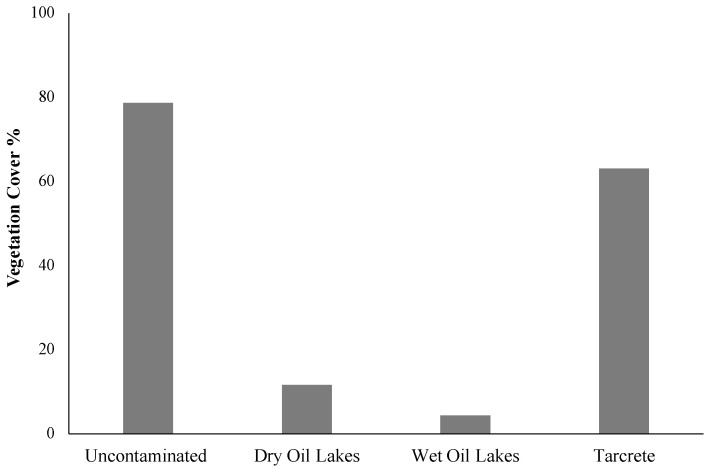
Vegetation cover distribution among three TPH-contaminated sites.

The results of the regression analysis between TPH and soil parameter contents showed that TPH contamination significantly influenced soil parameter concentration, which differed between the three different types of oil contamination. The impact of TPH on soil properties differed according to the various TPH categories, as shown in Figure 4. Most soil parameters were correlated with TPH concentration in the DOL, including EC, total Cr, Cr III, Pb, Zn, and Ba (Table 4). Forward stepwise regression analysis demonstrated that the best-fit model included EC, total Cr, Cr III, Zn, V, and Ba (*R*^2^ = 0.28, *p* < 0.01), showing relatively high concentrations of soil parameters when compared with the remaining soil parameters.

For WOL, all soil parameters were correlated with TPH contamination except Pb and V, showing a higher concentration of the correlated parameters. However, forward stepwise regression analysis showed that the best-fit model included soil pH, Cu, and V (*R*^2^ = 0.3, *p* < 0.01). According to the ANOVA test, a significant increase in concentration was detected in soil salinity, moisture, EC, and B in the WOL. The concentration in the DOL was similar to that in WOL in terms of B, Ni, Zn, total Cr, Cr III and V, showing a higher concentration compared with tarcrete and uncontaminated soils, which were significantly lower in these soil parameters. 

The results showed that in tarcrete-contaminated sites, TPH only correlated with EC (*R*^2^ = 0.37, *p* < 0.01). The ANOVA test showed that pH, Cu, and Pb were significantly higher at sites contaminated with tarcrete than in other sites. Additionally, the results of the regression analysis showed that all three TPH categories were correlated with EC, showing a significantly higher EC in WOL than that in other sites. No significant differences were found between DOL and tarcrete sites with respect to soil EC. In both DOL and WOL, TPH was correlated with V, which had higher concentration in both sites than that in tarcrete-contaminated and bare soils.

Generally, soil parameters did not show many significant differences between surface and subsurface layers among the three TPH-contaminated categories. Only moisture and salinity in WOL changed significantly between the surface and subsurface layers. In DOL, the concentration of most soil parameters was not significantly different between the surface and subsurface layers with the exception of salinity, aliphatic aromatics C35–C90, and Ni in the surface soils. For tarcrete-contaminated sites, only soil salinity showed significant differences between surface and subsurface layers.

### 3.3. Influence of Oil Properties and TPH on Vegetation Coverage

The results indicated that the vegetation cover was high in 2019. However, the distribution of vegetation cover differed among the three types of oil contamination (Figure 4). The highest vegetation cover was present covering the uncontaminated sites, where it reached 78%. At the contaminated sites, vegetation cover was highly present at the oil tarcrete-contaminated sites, covering 63% of such sites. WOL and DOL showed the lowest presence of vegetation cover, with vegetation covering 11% of the DOL-contaminated sites, followed by 4% vegetation cover at WOL-contaminated sites. High vegetation cover was associated with the high rainfall season in 2019.

## 4. Discussion

The results showed that TPH concentration differed between the three TPH-contamination categories, which highly influenced the concentrations of soil parameters and impacted the growth of native desert plants. Vegetation cover was negatively impacted by the high concentration of TPH at WOL and DOL sites, which was more likely related to the significant increase in soil parameter concentrations. Low vegetation cover and distribution at WOL and DOL sites could be associated with the high concentrations of heavy metals, higher than 50 mg/kg for Ba, and 10 mg/kg for V, Zn, Ni, Cr at these sites. Wood Jr and Nash III [23] showed that the concentration of Cu, Cd, Pb, Fe, and Zn in the soil highly impacts the growth of native desert plants, including annuals, perennials, grasses, cacti, and some shrubs. It has been illustrated by previous studies that heavy metals negatively affect many ecological processes, as they reduce photosynthetic efficiency, leading to a reduction in plant growth. These heavy metals negatively impact chlorophyll biosynthesis and the electron transport system in photosystems I and II [24]. Such effects can clearly explain the significant damage to native desert plants at WOL and DOL sites. 

Tarcrete locations showed higher vegetation coverage, at levels similar to the uncontaminated sites. The presence of high vegetation cover at tarcrete sites could be related to several factors. The results indicated that tarcrete-contaminated sites had lower concentrations of TPH and soil parameters, especially Ba, V, Zn, Ni, Cr metals, compared with WOL and DOL sites. The concentration of most soil parameters was similar to that in the uncontaminated sites, which could be the main reason for vegetation-cover support, although Pb and Cu were present in higher concentrations at the tarcrete sites compared with the other categories of contaminated site. This could be related to the degree of pollutants, as interactions between pollutants may occur when plants are exposed to more than one heavy-metal pollutant [25]. Synergistic interactions between heavy metals and plants are common and very important to ecological processes in natural ecosystems, since pollution is caused by more than one pollutant in many polluted environments [26]. High Pb concentrations can destroy the initial aboveground parts of the plant; however, roots are not affected, which supports the regrowth of shoots in several cases [26]. This could be one of the main reasons for the higher levels of growth of native desert plants at tarcrete sites, since these sites had been disturbed for a long period (since 1991).

The results also showed that the soil moisture content at the subsurface layer in tarcrete-contaminated sites was higher compared with that of the surface soils, which could positively support vegetation growth at these sites. This finding indicates that the hard layer of tarcrete on the surface is acting as mulch, reducing evaporation and evapotranspiration rates, which allows the accumulation of more moisture content available for plant uptake [27]. Field assessments conducted by KNFP showed that plant species started to grow significantly within tarcrete crakes. However, sites in which tarcrete layers were removed did not show any plant regrowth, which is likely related to the removal of topsoil during the cleaning operation, impacting soil nutrients in the surface soils.

The presence of native plants within the contaminated sites is influenced by the type of plant, namely, whether they are perennials or annual plants. This is important since perennial shrubs can accumulate high amounts of heavy metals due to the larger size of the shrubs compared with tiny annual grasses [28]. Native plants can accumulate heavy metals such as Pb, As, Zn, Cu, Cd, Ni, Cr, and Fe in the range of critical and phytotoxic values, particularly in the roots, depending on the type of plant [29]. A previous study showed that *Haloxylon salicornicum*, *Cyperus conglomeratus* and *Rhanterium epapposum* are the dominant perennial species found over most hydrocarbon contaminated sites. However, it is essential to consider precipitation and soil types in the ecological succession in arid ecosystems [30]. Previous studies have illustrated that most vegetation cover detected in Kuwait deserts consists of annual plants that appear only after high rainfall seasons, providing considerable variation in plant coverage from one year to another [31]. However, perennial plants are limited, as they are presented in protected and uncontaminated fenced sites where anthropogenic activities are not present [32]. The succession process of vegetation survival and regrowth over contaminated soils also depends on the type of soil, with different soil types supporting particular plant species. Vegetation secession could also be associated with accumulated layers of clean sediment covering the contaminated layers. Thus, further research is required on the mechanisms of native desert plants in order to develop a better understanding of the resiliency of native desert plants to various levels of TPH and heavy metal concentrations. 

On the basis of this study’s findings, it can be asserted that tarcrete-contaminated sites are more suitable for the regrowth of native desert plants than WOL and DOL sites. These sites are also perfect for restoration and revegetation, since they are the most common type of contaminated site at the Al-Burgan oil field and in other contaminated areas, including the Um Gudair oil field. The contamination level of TPH and soil parameters present at the tarcrete sites could also be used as an indirect indicator to determine the optimal contamination level for vegetation succession.

## 5. Conclusions

The outcomes of this work help build an understanding of the resiliency of native plants subjected to different concentrations of pollution at various TPH-contaminated sites, and provide information on the optimal type of TPH-contaminated site that can support the regrowth of native plants. The study provided an overall analysis of the resiliency of native desert vegetation following three major types of TPH contamination, namely, WOL, DOL, and tarcrete contamination. It showed the success of vegetation regrowth over tarcrete-contaminated sites, indicating that these are the most suitable type of site for the growth of native desert plants. More research is needed to understand the resiliency of major perennial natives to the three types of hydrocarbon contamination, to provide further insight into the native plants’ phytoextraction potential and more efficient form of phytoremediation. It is also vital to build knowledge of the quantitative relationships of the processes that occur within natural systems in arid landscapes, to better understand the mechanisms by which environmental factors influence the regrowth of desert vegetation, paving the way for ecosystem resiliency.

## Figures and Tables

**Table 1:** Number of samples, sampling methods, and sampling depth for the different categories of contaminated sites.

Sites	Surface Layer	Subsurface Layer	Deep Layer	Number of Samples	Method of Sampling
Dry Oil Lake	Dry-S (22 samples) 0.0–0.15 m	Dry-D1 (19 samples) 0.16–0.40 m	Dry-D2 (10 samples) >0.41 m	51	Grab method
Wet Oil Lake	Wet-S (11 samples) 0.0–0.30 m	Wet-D1 (16 samples) 0.31–0.65 m	Wet-D2 (7 samples) >0.65 m	34	Core method
Tarcrete	Tar-S (22 samples) 0.01–0.05 m	Tar-D (13 samples) 0.10–0.15 m	No samples were collected	35	Grab method
Bare Soil	BS-S 0.0–0.05 m	No samples were collected	No samples were collected	25	Grab method

**Table 2:** List of analyzed parameters for collected soil samples.

	Type of Parameter	Preparation Method	Analysis Method
pH	Chemical properties	USEPA 9045	USEPA 9045
Moisture Content (%)	Indicator of quality and fertility of soil	ASTM D2216	ASTM D2216
Salinity (SAR)	Chemical properties	USEPA 6010 B	USEPA 6010 B
Electrical conductivity (µS/cm) (EC)	Chemical properties	USEPA 9050 A	USEPA 9050 A
Aliphatic Aromatics C35–C90 (mg/kg)	Chemical compounds	USEPA 8260	USEPA 8260
TPH (HEM) (mg/kg)	Chemical compounds	USEPA 9071 B	USEPA 9071 B
Chromium III (mg/kg) (Cr)	Heavy metals	USEPA 3015 B	USEPA 6010 B
Total Chromium (mg/kg) (Cr)	Heavy metals	USEPA 3015 B	USEPA 6010 B
Copper (mg/kg) (Cu)	Heavy metals	USEPA 3015 B	USEPA 6010 B
Nickel (mg/kg) (Ni)	Heavy metals	USEPA 3015 B	USEPA 6010 B
Lead (mg/kg) (Pb)	Heavy metals	USEPA 3015 B	USEPA 6010 B
Zinc (mg/kg) (Zn)	Heavy metals	USEPA 3015 B	USEPA 6010 B
Vanadium (mg/kg) (V)	Heavy metals	USEPA 3015 B	USEPA 6010 B
Water-soluble Boron (mg/kg) (B)	Nutrient	USEPA 3015 B	USEPA 6010 B
Barium (mg/kg) (Ba)	Heavy metals	USEPA 3015 B	USEPA 6010 B

**Table 3:** Results of soil-parameter analysis (minimum, maximum, average, and standard deviation) in the four categories. MAX, maximal value; MIN, minimal value; AVG, average value; and STDEV, standard deviation.

	DOL				WOL				Tarcrete				Bare Soil (Uncontaminated)			
	MAX	MIN	AVG	STDEV	MAX	MIN	AVG	STDEV	MAX	MIN	AVG	STDEV	MAX	MIN	AVG	STDEV
pH	8.7	6.9	7.7	0.3	8.3	6.5	7.2	0.4	8.9	7.4	8.1	0.4	8.6	7.5	7.9	0.3
Moisture (%)	9.2	0.2	1.4	1.5	28.7	1.3	7.7	6.3	2.6	0.5	1.3	0.5	2.3	0.5	1.1	0.5
Salinity (SAR)	8.7	0.9	3.2	1.8	16.3	2.4	7.7	2.6	4.6	0.9	2.4	0.9	1.8	0.8	1.3	0.3
Electrical conductivity (µS/cm)	8950	173	2291	2323	32,560	1400	15,634	7847.9	5213	168	1312.3	1105.3	624.5	125	349.1	163
Aliphatic Aromatics C35–C90 (mg/kg)	162,354	176	40,059	40,406	586,100	626	97,776	129,882.9	131,680	0.5	16,746.2	30,795.5	2658	1	988.7	779.9
TPH (mg/kg)	146,119	138	35,741	36,347	527,490	563	87,961	116,916.3	128,541	135	2,4063.3	31,014.4	1685	186	726.8	404.3
Chromium III (mg/kg)	67.7	7.2	21.6	13.5	27	7.8	14.9	4.9	26.5	0.2	11.2	8.4	23.5	0.2	10.5	8.1
Chromium Total (mg/kg)	67.5	7.2	21.6	13.4	27	7.5	14.9	4.9	26.5	0.2	11.2	8.4	23.5	0.2	10.5	8.1
Copper (mg/kg)	15.6	1.2	4.6	3.2	6	1.7	3.7	1.2	13.6	1.4	7.1	3.9	12.1	1.1	4.6	2.9
Nickel (mg/kg)	28.9	2.2	13.8	7.8	24	5.6	13.6	4.4	15.8	1.9	6.2	3.8	12.2	1.6	6.3	3.5
Lead (mg/kg)	7.5	0.9	2.4	1.5	4	0.8	1.9	0.7	9.4	0.8	3.9	2.7	10.3	0.9	2.5	2.1
Zinc (mg/kg)	23.5	4.9	14.7	4.6	21	4.1	11.9	4.2	16.5	1.2	6.9	52	16.2	1.1	8.3	5.1
Vanadium (mg/kg)	32.5	2.3	12.9	8.3	26	5.6	13.3	5.7	11.2	0.2	4.9	3.6	16.7	0.2	5.5	4.5
Water-soluble boron (mg/kg)	23.5	1	5.7	5.4	10	1.5	5.2	2.1	2.8	0.2	1.4	0.8	2.7	0.2	1	0.8
Barium (mg/kg)	172.6	18.1	62.8	33.3	154	33.8	105.1	31.5	65.3	0.2	34.8	20.5	57.7	0.2	29	17.5

**Table 4:** Summary of regression analysis results between TPH and soil parameters in three TPH-contaminated sites.

Independent Parameters	(DOL)					(WOL)					Tarcrete				
	*R* ^2^	RMSE	*p* Value	*R*^2^-Added	*p* Value	*R* ^2^	RMSE	*p* Value	*R*^2^-Added	*p* Value	*R* ^2^	RMSE	*p* Value	*R*^2^-Added	*p* Value
	Simple Linear Regression			Multivariate, Stepwise		Simple Linear Regression			Multivariate, Stepwise		Simple Linear Regression			Multivariate, Stepwise	
pH	0.02	36,416	0.371			0.31	98,927.3	<0.001 *	0.31	<0.001 *	0.032	31,054	0.351		
Moisture %	0.05	35,734	0.121			0.01	118,083	0.558			0.051	30,612	0.182		
Electrical conductivity (µS/cm)	0.27	31,309	<0.001 *	0.27	<0.001 *	0.13	111,089	0.041 *			0.371	24,974	<0.001 *	0.37	<0.001 *
Chromium III (mg/kg)	0.16	33,586	0.003 *	0.07	0.031 *	0.22	105,157	0.006 *			0.001	31,471	0.891		
Chromium Total (mg/kg)	0.16	33,696	0.004 *	0.06	0.033 *	0.22	105,173	0.006 *			0.001	31,471	0.891		
Copper (mg/kg)	0.06	35,503	0.071			0.29	100,081	0.001 *	0.29	0.001 *	0.031	30,998	0.323		
Nickel (mg/kg)	0.02	36,429	0.382			0.13	110,828	0.037 *			0.012	31,356	0.612		
Lead (mg/kg)	0.14	33,951	0.006 *			0.09	113,029	0.078			0.051	30,663	0.192		
Zinc (mg/kg)	0.25	31,903	<0.001 *	0.25	<0.001 *	0.17	108,311	0.016 *			0	31,480	0.981		
Vanadium (mg/kg)	0.01	36,528	0.481	0.06	0.043 *	0.04	116,206	0.245	0.16	0.006 *	0	31,480	0.991		
Water-soluble boron (mg/kg)	0.01	36,469	0.421			0.12	111,326	0.044 *			0.031	30,942	0.293		
Barium (mg/kg)	0.1	34,814	0.023 *	0.05	0.045 *	0.16	109,104	0.021 *			0.022	31,230	0.472		

(* represents the significant variables according to the statistical test).

## Data Availability

The data presented in this study are available on request from the corresponding author. The data are not publicly available due to the restrictions of Kuwait Oil Company (KOC).
